# Emergency medical dispatchers’ ability to identify large vessel occlusion stroke during emergency calls

**DOI:** 10.1186/s13049-021-00914-1

**Published:** 2021-07-19

**Authors:** Pauli E. T. Vuorinen, Jyrki P. J. Ollikainen, Pasi A. Ketola, Riikka-Liisa K. Vuorinen, Piritta A. Setälä, Sanna E. Hoppu

**Affiliations:** 1 Emergency Medical Services, Centre for Prehospital Emergency Care, Pirkanmaa Hospital District, Tampere University Hospital, PO Box 2000, FI-33521 Tampere, Finland; 2 Department of Neurosciences and Rehabilitation, Tampere University Hospital, Tampere, Finland; 3 Faculty of Medicine and Health Technology, University of Tampere, Tampere, Finland

**Keywords:** Emergency medical dispatch, Emergency medical services, Large vessel occlusion stroke

## Abstract

**Background:**

In acute ischemic stroke, conjugated eye deviation (CED) is an evident sign of cortical ischemia and large vessel occlusion (LVO). We aimed to determine if an emergency dispatcher can recognise LVO stroke during an emergency call by asking the caller a binary question regarding whether the patient’s head or gaze is away from the side of the hemiparesis or not. Further, we investigated if the paramedics can confirm this sign at the scene. In the group of positive CED answers to the emergency dispatcher, we investigated what diagnoses these patients received at the emergency department (ED). Among all patients brought to ED and subsequently treated with mechanical thrombectomy (MT) we tracked the proportion of patients with a positive CED answer during the emergency call.

**Methods:**

We collected data on all stroke dispatches in the city of Tampere, Finland, from 13 February 2019 to 31 October 2020. We then reviewed all patient records from cases where the dispatcher had marked ‘yes’ to the question regarding patient CED in the computer-aided emergency response system. We also viewed all emergency department admissions to see how many patients in total were treated with MT during the period studied.

**Results:**

Out of 1913 dispatches, we found 81 cases (4%) in which the caller had verified CED during the emergency call. Twenty-four of these patients were diagnosed with acute ischemic stroke. Paramedics confirmed CED in only 9 (11%) of these 81 patients. Two patients with positive CED answers during the emergency call and 19 other patients brought to the emergency department were treated with MT.

**Conclusion:**

A small minority of stroke dispatches include a positive answer to the CED question but paramedics rarely confirm the emergency medical dispatcher’s suspicion of CED as a sign of LVO. Few patients in need of MT can be found this way. Stroke dispatch protocol with a CED question needs intensive implementation.

## Background

In acute ischemic stroke (AIS) due to a large vessel occlusion (LVO), two million neurons perish every minute the artery remains blocked [1]. In high-priority dispatches, only few patients truly benefit from high-speed driving with lights and sirens, considering that this type of driving endangers the common traffic flow [2]. AIS is the one where every single minute counts [3, 4] and even some overtriage is considered acceptable to achieve time critical interventions in time [5].

Extensive measures have been implemented to reduce the in-hospital delays of door-to-treatment-time to recanalise the occluded artery by means of pharmacological thrombolysis or endovascular mechanical thrombectomy (MT) of LVO [6, 7]. Despite such efforts, prehospital delays reduce the likelihood of achieving the best post-AIS outcome. Continual stroke awareness campaigns directed at the public are needed to decrease onset-to-call times [8]. It takes only a median of 3 min after receiving the emergency call to dispatch the emergency medical services (EMS) [9], but dispatching the EMS with a correct suspicion of stroke increases the probability of the paramedics recognising an actual stroke case [10]. When the paramedics suspect stroke, they are more likely to give the appropriate prenotification to the receiving hospital, which in turn decreases the in-hospital delay [11].

Endovascular treatment of LVO with or without thrombolysis has been shown to be more effective than thrombolysis alone but this intervention is available only in comprehensive stroke centres [12]. The AIS management guidelines of the American Heart Association and American Stroke Association encourage the seeking of accurate methods to distinguish AIS without LVO from AIS caused by LVO by the paramedics already at the scene [13]. A multitude of strategies with differing performances are available to detect LVO, but few have been validated in true prehospital use [14].

We have previously introduced a LVO scale for prehospital use utilising a binary question regarding whether the patient’s head or gaze is away from the side of the hemiparesis, i.e. conjugated eye deviation (CED) question [15]. Reports on the emergency center’s ability to detect large vessel occlusion have not been published. A suspicion of LVO noted already during the emergency call could change the EMS’ stroke protocol. For example, sending a mobile stroke unit to the scene would give a definitive answer regarding whether the patient should be directed primarily to a comprehensive stroke centre bypassing the primary stroke centre [16]. Activation of a helicopter EMS unit to remote missions with suspected LVO could be a valuable method to decrease prehospital delays before LVO recanalization [17].

Our aim was to investigate how the CED question is answered in emergency call processing**.** We put further emphasis on reporting whether the paramedics concurred with a positive CED answer in the emergency call and defining the characteristics of patients dispatched with suspected LVO in the city of Tampere, Finland. Finally, we aimed to find out what is the proportion of the emergency medical dispatcher’s suspicion of CED among all patients treated with MT.

## Methods

### Setting

Tampere is the third biggest city in Finland, with about 230,000 inhabitants, and is the central city of the Pirkanmaa hospital district. The emergency department (ED) of Tampere University Hospital serves as the only ED for the city’s residents and as a comprehensive stroke centre for the hospital’s specific catchment area encompassing a population of about 900,000. The ED has up to 100,000 patient admissions yearly and there are 20–30 physicians on call depending on the shift. The hospital has an on-call neurologist 24/7 and an interventional radiologist performing approximately 200 MT yearly. All EMS missions in the city of Tampere are operated by the Tampere region rescue department. The yearly number of EMS missions in the city of Tampere is approximately 30,000. The ambulances sent to all suspected stroke dispatches are staffed with an advanced life support trained nurse paramedic, with a degree from the university of applied sciences for health care. The nurse paramedic has a working partner with a similar education or an emergency medical technician.

Puolakka et al. [9] describe in detail the emergency call processing used prior to 2018 for suspected stroke cases in Finland. The emergency call processing for stroke is congruent with the Medical Priority Dispatch System card #28 [18]. The Finnish Emergency Response Centre Agency renewed their entire emergency response system to a computer-aided system named Emergency Response Integrated Common Authorities (ERICA). When ERICA was introduced (over the years 2018 and 2019), the face-arm-speech triad or the caller’s suspicion of a stroke, with the symptoms’ onset time (6 h as the equator) were accompanied by the CED question regarding whether the patient’s face or gaze is turning away from the side of the hemiparesis or not. The dispatcher may answer this question with ‘no’, ‘yes’ or ‘unknown’. The stroke dispatch’s priority is unaffected by the answer, but a positive answer generates an additional notification to the EMS field commander. The dispatcher may also leave the CED question unanswered during the call.

### Study design

We retrospectively reviewed all stroke dispatches in the city of Tampere from the 13 February 2019 until 31 October 2020.

### Data collection

We verified the answer to the CED question from the ERICA reports. The patient data from stroke dispatches with a positive answer to the CED question was obtained from the electronic patient records. From those EMS missions where the paramedics decided that the patient was not in need of ambulance transport to the ED, we retrieved a copy of the EMS report. We also went through the patient admission records of the Tampere University Hospital ED to establish the exact number of MT patients from the city of Tampere during the same period.

### Statistics

We used the spreadsheet software Microsoft Excel 2016 (Microsoft Corporation, Redmond, USA) to analyse the data. Descriptive continuous variables were expressed with medians and interquartile ranges. A chi-square test was used for categorical statistical comparison and a student’s t-test for continuous comparisons. A *p*-value of less than 0.05 was regarded as significant.

### Ethics

The Ethics Committee of the Tampere University Hospital supported the study design (ETL R20082R), and permission to view the patient records was given by the hospital’s medical director. Informed consent was deemed unnecessary due to the retrospective chart-review design of the study.

## Results

We found a total of 1913 EMS dispatches with dispatcher-suspected stroke, of which 1491 (78%) were high priority. The answer to the CED question was available for 1900 dispatches: answers were ‘no’, ‘yes’, and ‘unknown’ in 688 (36%), 81 (4%) and 810 (43%) dispatches, respectively, while in 321 (17%) dispatches, the question was unanswered.

Of the 81 cases with positive answers to the CED question gained during dispatch, 24 cases (30%) were diagnosed in the ED as AIS, four cases (5%) were diagnosed as spontaneous intracerebral haemorrhage, 10 cases were not transported by ambulance to the ED, and stroke was not identified in the remaining 43 cases (53%). Table 1 presents the characteristics of the 81 patients with a positive CED answer. Diagnoses set at the ED were symptom-based (ICD-10 R19.5–R55) in 13 patients of the whole group of 81. Twenty-one patients (26%) were discharged directly from the ED.

**Table 1:** Characteristics of patients with a positive answer to the conjugated eye deviation question (*n* = 81)

	n, (%), [Q1–Q3]
Median age, years	75 [62–90]
Gender male	36 (44)
Location of the EMS mission	
Private residence	48 (59)
Healthcare facility / nursing home / assisted living	22 (27)
Public location	11 (14)
Medical History^a^	
Hypertension	53 (65)
Diabetes	25 (31)
Atrial fibrillation	21 (26)
Dementia	21 (26)
Previous CVA	23 (28)
Existing advance care planning	17 (21)
Gaze deviation	
Verified by the paramedics	9 (11)
Verified in the ED	8 (10)
ED diagnosis	
AIS	24 (30)
treated with MT	2
Spontaneous ICH	4 (5)
Traumatic ICH	3 (4)
Epileptic seizure	6 (7)
Infection	5 (6)
Symptom-based diagnosis	13 (16)
Hypoglycaemia	2 (3)
Aftermath of CVA	3 (4)
Intoxication	3 (4)
Other	8 (10)
(traumatic brain injury, mydriasis, hypotension, kidney failure, arthritis, hydrocephalus, gluteal trauma, monitoring)	
No ED admission	10 (12)

^a^Described in the ED admission report

*AIS* Acute ischemic stroke, *CVA* Cerebrovascular accident, *ED* Emergency department, *EMS* Emergency medical services, *ICH* Intracerebral haemorrhage, *MT* Mechanical thrombectomy

**Fig. 1:**
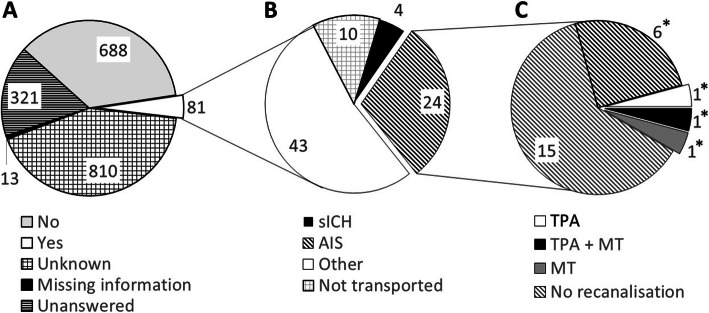
Chart diagram of patient flow. (n). **A** Distribution of different answers to the CED question among all the stroke dispatches in the city of Tampere (*n* = 1913). **B** Emergency department diagnoses of patients with dispatcher suspected eye deviation. **C** Final choice of recanalisation therapy (*Paramedic confirmed conjugated eye deviation). AIS: Acute ischemic stroke, MT: Mechanical thrombectomy, sICH: Spontaneous intracerebrebral haermmorhage, TPA: Tissue plasminogen activator

Figure 1 shows the proportions of the four different possible responses to the CED question and the proportions of the subgroups among the patients with a positive CED answer. Only 9 (11%) of the 81 patients with positive CED answers during the emergency call presented evident CED when the paramedics arrived at the scene. In one patient, CED abated during the transport. This patient was found to be the only one with incongruent CED presentation when the reports of the paramedics and the patient records were compared. Six of the true positive CED patients had AIS, one had spontaneous intracerebral haemorrhage and two had postictal Todd’s paresis. Two patients were treated with MT. One of the AIS patients with CED had no clear target for endovascular treatment and showed a good resolution of stroke symptoms with thrombolysis. This patient had previously had AIS years earlier, and this new stroke possibly worsened the clinical picture. It was considered that three AIS patients would not benefit from recanalization therapy due to severely disabling dementia, and therefore angiographic imaging was considered redundant.

Altogether, 21 patients with LVO and MT were transported from the city of Tampere to the ED by ambulance during the study period. This means that the dispatcher’s question about CED found 10% of patients in need of an immediate endovascular procedure.

During the follow-up there were 1065 CED questions with ‘yes’, ‘no’ or ‘unknown’ answers during day shifts and 514 during night shifts. The CED question was left unanswered 197 times during day shifts and 124 times during night shifts, meaning that the CED question was significantly more frequently left unanswered during night shifts than during day shifts (*p* = 0.036). The options ‘yes’, ‘no’, ‘unknown’ and ‘unanswered’ were evenly distributed within the shifts (data not shown).

## Discussion

This is the first study to report the effect of a stroke dispatch protocol with an added routine binary question about an evident cortical sign: conjugated eye deviation. The dispatcher got a positive answer to the CED question in 4% of the suspected stroke dispatches. Further analyses revealed that the paramedics observed true CED in 11% of these suspected LVO cases and that of all the MT patients from the city of Tampere only 10% were in this group of positive CED answers.

The only previous study to report the prevalence of confirmed LVO strokes among dispatcher- or paramedic-suspected strokes is the PLUMBER study [19]. Curiously, the rate of LVO prevalence reported in the PLUMBER study (4.9%) was similar to the proportion of positive answers to the CED question in our study (4%). Unfortunately, we found many false positives, and only a minority of the patients we truly wanted to find: patients in need of MT. Krebes et al. [20] also report visual disturbance including CED mentioned in 5% of emergency calls concerning stroke but do not report its association with LVO.

A great deal of research has been conducted concerning the emergency medical dispatcher’s sensitivity towards recognising stroke per se during an emergency call [21]. A stroke diagnosis is highly probable when the caller spontaneously mentions the word ‘stroke’ [22]. Studies have repeatedly called attention to the fact that false negative dispatch codes in stroke patients are often due to initial words mentioned by the caller in an emergency call such as ‘fall’, ‘sick person’ and ‘unconsciousness/confusion’ [22, 23]. We also believe that this explains why, in our study, the CED question found only a minority of patients being in need of MT. Patients with LVO collapse at the moment of arterial occlusion, they might have global aphasia or they present with a grave wake-up stroke. Therefore hemiparesis remains unnoticed. The ERICA protocols for an unconscious, a sick or a fallen person do not direct the dispatcher to ask whether there are stroke symptoms present, let alone about possible CED.

One could assume that a binary question about the direction of the patient’s gaze could be easily incorporated into the dispatch protocol for stroke. Yet in most of the dispatches in our study, the answer to the CED question was ‘unknown’ or the question was unanswered. There are a number of possible explanations for this. First, the protocol was updated at the same time as the introduction of an entirely different, computer-aided emergency response system. It is impossible to describe exactly the process of implementing the CED question before deploying ERICA. During the first months after ERICA’s deployment, the dispatchers expressed their frustration about the increased workload and problems in implementing the new system. These circumstances may have been a factor in the variation of the unanswered CED questions between different shifts.

Second, few emergency medical dispatchers in Finland are healthcare professionals and have not necessarily understood the importance of prehospital LVO recognition. Third, a positive answer to the CED question did not visibly influence the stroke dispatch itself or its priority from the dispatcher’s perspective because it only generated an additional notice for the EMS field commander. Compared to the universally known Face Arm Speech, CED is not a commonly identified stroke symptom. An average bystander would be unlikely to mention it spontaneously on the phone.

Fourth, if the emergency caller is an anxious family member of the LVO patient, the caller might not be willing to raise the distressed patient’s eyelids to look at the patient’s eyes.

Lawner et al. [24] emphasized the importance of extensive training of paramedics to improve the accuracy of the LVO scale. High-quality implementation is also needed for emergency dispatch protocols that aim to identify suspected LVO.

The study raises more questions than it answers. While we learned that a simple binary question about the direction of the stroke patient’s gaze is not a feasible way of detecting LVO, we still do not know how to positively identify LVO in patients during the emergency call. We also did not come to a definitive conclusion as to why only a minority of responses to the CED questions were ‘yes’ or ‘no’ (as opposed to ‘unknown’ or the question remaining unanswered). We wonder how there can be 72 patients in whom the caller gave a positive answer to the CED question but the paramedics didn’t perceive CED at the scene. We cannot confirm whether the CED had subsided before ambulance arrival, the caller answered the CED incorrectly or the dispatcher marked the answer incorrectly to the ERICA report. To investigate this further we would need to analyse the emergency call recordings.

### Strengths and limitations

The single-centre design is this study’s greatest strength. The study population comprises the residents of a single city. Emergency calls are handled by a national emergency response centre using a strict computer-based protocol. Every stroke dispatch in this study was operated by one EMS agency, and every patient transported was taken to the same university hospital ED. All of these eliminate the bias multiple operators could produce in different phases of care. We are not aware that any other emergency response centre or EMS agency has yet attempted to identify LVO during the emergency call.

Due to the retrospective design there is a minute possibility that there are additional EMS missions where the dispatcher first began with the stroke dispatch protocol and answered the CED question but then decided to use e.g. an ‘unconscious person’ dispatch code. These cases would not have come up in our search since we reviewed only EMS missions with suspected stroke dispatch as the final dispatch code.

This study was not determined to investigate differences in the prevalence of LVO in patients across the different possible responses given by emergency callers to the CED question. A computed tomography angiography was performed only on patients with serious stroke symptoms and who were thought likely to benefit from MT. We did not further investigate the group of patients with a ‘no’ answer to the CED question, and hence we are unable to extract a figure for specificity or for a negative predictive value. All these limitations point to the need to further study the contents of the emergency calls involving LVO patients.

## Conclusion

An emergency medical dispatcher suspects CED as a sign of LVO in 4% of stroke dispatches, but only 10% of patients in need of MT can be found this way. Our study could not show that mobile stroke unit or helicopter EMS is worth dispatching with a CED question. However, in the future, the potential of emergency response centres in identifying LVO is worth exploring. The studies must pay attention to the simplicity and proper implementation of the set of questions.

### Data sharing

Deidentified data will be preserved for 10 years after publication. Proposals for data sharing should be directed to the corresponding author by email.

## Abbreviations

AIS: Acute ischemic stroke
CED: Conjugated eye deviation
ED: Emergency department
EMS: Emergency medical services
ERICA: Emergency Response Integrated Common Authorities
LVO: Large vessel occlusion
MT: Mechanical thrombectomy
